# Rapid Recovery of Cyanobacterial Pigments in Desiccated Biological Soil Crusts following Addition of Water

**DOI:** 10.1371/journal.pone.0112372

**Published:** 2014-11-06

**Authors:** Raeid M. M. Abed, Lubos Polerecky, Amal Al-Habsi, Janina Oetjen, Marc Strous, Dirk de Beer

**Affiliations:** 1 Sultan Qaboos University, College of Science, Biology Department, Al Khoud, Sultanate of Oman; 2 Max-Planck Institute for Marine Microbiology, Bremen, Germany; 3 MALDI Imaging Laboratory, University of Bremen, Bremen, Germany; 4 Department of Geoscience, University of Calgary, Calgary, Alberta, Canada; NERC Centre for Ecology & Hydrology, United Kingdom

## Abstract

We examined soil surface colour change to green and hydrotaxis following addition of water to biological soil crusts using pigment extraction, hyperspectral imaging, microsensors and ^13^C labeling experiments coupled to matrix-assisted laser desorption and ionization time of flight-mass spectrometry (MALD-TOF MS). The topsoil colour turned green in less than 5 minutes following water addition. The concentrations of chlorophyll *a* (Chl *a*), scytonemin and echinenon rapidly increased in the top <1 mm layer while in the deeper layer, their concentrations remained low. Hyperspectral imaging showed that, in both wet and dehydrated crusts, cyanobacteria formed a layer at a depth of 0.2–0.4 mm and this layer did not move upward after wetting. ^13^C labeling experiments and MALDI TOF analysis showed that Chl *a* was already present in the desiccated crusts and *de novo* synthesis of this molecule started only after 2 days of wetting due to growth of cyanobacteria. Microsensor measurements showed that photosynthetic activity increased concomitantly with the increase of Chl *a*, and reached a maximum net rate of 92 µmol m^−2^ h^−1^ approximately 2 hours after wetting. We conclude that the colour change of soil crusts to green upon water addition was not due to hydrotaxis but rather to the quick recovery and reassembly of pigments. Cyanobacteria in crusts can maintain their photosynthetic apparatus intact even under prolonged periods of desiccation with the ability to resume their photosynthetic activities within minutes after wetting.

## Introduction

Cyanobacteria are ubiquitous in all arid and semi-arid biological soil crusts (referred as crusts hereafter), where they play a vital role in fixing carbon and nitrogen, stabilizing the soil as well as altering the hydrological properties (e.g. water retention) of crust-covered soils [1]–[4]. The dominance of cyanobacteria in arid deserts is indicative of their eco-physiological adaptability to high temperature, UV radiation and desiccation. Whereas prolonged periods of desiccation lead to the oxidation and destruction of proteins, nucleic acids and cell membrane components of microorganisms [5], cyanobacteria in crusts have developed a variety of strategies to deal with water scarcity and to respond rapidly to sporadic rehydration during raining events [6]–[11]. The crust-forming cyanobacterium *Microcoleus vaginatus* was found to remain in a metabolically dormant state below the soil surface for extended periods during acute water deficiency, but rapidly resuscitate during brief periods of hydration [12], [13]. Desiccation-tolerant cyanobacteria have also been shown to regulate their intracellular water potential through the accumulation of compatible solutes such as trehalose and sucrose and to increase water retention through the production of exopolysaccharides [5], [14]. Recently, a detailed transcriptomic study revealed a rapid induction of genes related to oxidative and osmotic stress responses and to the synthesis of C and N storage polymers when crusts were exposed to desiccation [15].

The active upward and downward locomotory movement of cyanobacteria in benthic environments has been reported as an essential mode of survival in response to fluctuations in UV and light intensity, chemical concentration and salinity [16]–[20]. *Microcoleus chthonoplastes* was shown to migrate a distance of 0.25 mm and 1–2 mm when exposed to high UV-B radiation and high salinities, respectively [16], [17], whereas *Spirulina* and *Oscillatoria* migrated a distance of 0.6–1 mm when exposed to different combinations of UV and visible radiation [20]. Cyanobacteria have been shown to migrate a distance of 1.5–2 mm upwards to track water in crusts, a process termed hydrotaxis, leading to the greening of the crust surface after wetting [12], [21]. While water addition to dry crusts from a desert in Navarre, NE Spain resulted in an evident change in the surface colour to green within a few minutes, this greening occurred in several hours in crusts from Colorado Plateau (USA) [21]. Our field and laboratory observations with crusts from the deserts of Oman revealed a similar greening phenomenon of the surface within minutes after water addition. The different time constants of greening in different crusts suggest different mechanisms. Indeed, since the description of hydrotaxis in crustal cyanobacteria [12], [21], very few studies were performed to find out if this phenomenon occurs in other crusts and if hydrotaxis is widespread among other cyanobacterial species.

We combined photopigment detection by high performance liquid chromatography (HPLC) and hyperspectral imaging, detection of *de novo* synthesized ^13^C labeled Chl *a* using MALDI-TOF MS and oxygen microsensors to investigate the colour change and start-up of photosynthesis upon wetting of crusts from the Sultanate of Oman. These crusts were previously studied with respect to their bacterial diversity, pigment composition and nitrogen fixation capabilities [22]. The main objective of the current study was to assess whether hydrotaxis is the mechanism of the very rapid greening in crusts from Oman, or if another mechanism is involved.

## Materials and Methods

### Crust samples and bacterial community composition

Crust samples were collected from a desert region in Wadi Al Khoud, near Muscat, northern Oman (23°4.595 ″N, 58 °9.055 ″E). No specific permission was required to access the site. The field studies did not involve any endangered or protected species. The site is surrounded by low limestone hills with few sporadically distributed trees of *Acacia tortilis* in the area. Crust pieces with flat surfaces (ca. 24 cm^2^) and approximately 1 cm thickness were collected from the topsoil in areas beneath the canopy of an *Acacia tortilis* tree. Samples were stored in plastic boxes and kept dry in the dark until analysis.

The bacterial community composition in the crusts was revealed by bacterial tag-encoded FLX amplicon pyrosequencing (bTEFAP) using the GS FLX titanium sequencing kit XLR70 according to the MR DNA protocols (www.mrdnalab.com). Sequences were cleaned and analysed as previously described [23]–[24]. Additionally, cyanobacterial filaments dominating the crust's surface were picked up after water addition using sterile forceps under a dissecting microscope. The filaments were sequenced using cyanobacteria-specific primers and the sequences were analysed using the ARB software as previously described [16].

### Wetting experiments and pigment analysis

Wetting experiments were performed in sterile Petri dishes by adding distilled water to crust pieces until saturation. These experiments were carried out under illumination and in the dark, in inverted crust pieces and by wetting the pieces from top, bottom and sides. Pigments were extracted from the top (<1 mm) and bottom (1–10 mm) layer of the crusts and measured using HPLC at different time intervals after wetting. Pigment analysis was performed to find out if the observed greening of the crust surface was due to the upward movement of cyanobacteria (i.e. hydrotaxis), judged by the increase in cyanobacterial pigments in the top layer and their decrease in the bottom layer. The top layer of the crust (100–150 mg) could be easily peeled off using a sterile scalpel as it was visibly well defined. Pigments were also extracted from dry crusts as well as from pieces directly after wetting. Extraction of the pigments was performed under dim light with ice-cooled 90% acetone after sonication and incubation at −20°C for 24 h. The supernatants were filtered through 0.45 µm Acrodisc CR 4 mm syringe filter (Pall Gelman Laboratory, USA). The pigments were analyzed using reverse phase high performance liquid chromatography (HPLC) that consisted of a Waters 996 photo diode array detector (PDA) and a Waters 2690 separation module (Waters, Massachusetts, USA). A 125×4.6 mm vertex column packed with Eurospher-100 C18 of 5 µm particle size was used (Knauer GmbH, Berlin, Germany). The pigments were identified by comparing the retention time and the spectrum with commercially available pigment standards (from DHI Water and Environment, Denmark, Sigma-Aldrich and Merck, Germany).

### Hyperspectral imaging

Hyperspectral imaging [25] was used to characterize the temporal changes in the distribution of pigments in the crusts following the addition of water. Flat crust pieces were fixed on a white standard substrate (Spectralon: Labsphere, USA) using modelling clay and placed in a Petri dish. Their orientation was horizontal and vertical to allow measurements from the top and from the side, respectively. Imaging was also performed on very thin crust pieces (ca. 0.5 mm) after removing the soil beneath. Illumination was provided by a halogen lamp (Philips), which allowed determination of reflectance in the spectral range of 400–900 nm with a spectral resolution of ∼1 nm. The cyanobacterial-specific pigments Chl *a* and phycocyanin were quantified using the second derivative of the reflectance spectrum at wavelengths corresponding to their *in vivo* absorption maximum (673 nm for Chl *a* and 622 nm for phycocyanin). Although this measurement was not strictly quantitative, it provided reliable and non-destructive estimation of the relative changes in the pigment concentrations [25].

### Detection of ^13^C labeled Chl *a* using MALDI-TOF

^13^C labeling experiment of the cyanobacterial pigments was carried out to distinguish between the existing and the *de novo* synthesized molecules of Chl *a*. Triplicate samples (0.5 g each) of intact crust pieces were incubated in 3 ml of freshly prepared 10 mM ^13^C-labeled bicarbonate solution in 5 ml vials. The vials were covered with a transparent foil prior to wetting to avoid loss of the label through equilibrium with the air. The incubations were carried out at 26°C under illumination. Crust pieces were sampled at different time points (0, 0.5, 1, 2, 4, 8, 24, 48 and 72 h), deactivated in liquid nitrogen and kept at −20°C until analysis. To measure ^13^C bicarbonate, 0.5 ml of 50 mM boiled Tris buffer was added to the crust pieces, vortexed and then centrifuged for 10 minutes at 14,600 rpm. One half ml of the supernatant was injected into an exetainer containing 1 ml acetate buffer (pH = 4), preflushed with N_2_ gas. The exetainers were shaken every 15 minutes for 2 h and then kept inverted until analysis by mass spectrometry. Pigments from crusts were extracted with ice-cooled 90% acetone (HPLC grade) as previously described [26]. The pigments were analyzed using MALDI-TOF mass spectrometry (autoflex speed, Bruker Daltonics, Bremen). The pigment extract in acetone (i.e. 1 µl) was spotted onto a MALDI-TOF stainless steel target plate and mixed with 1 µl of matrix solution (7 mg/ml alpha-Cyano-4-hydroxycinnamic acid (HCCA) in 50% Acetonitrile (ACN) and 0.2% trifluoro acetic acid (TFA)). Spectra were acquired using flexControl software version 3.3 (Bruker Daltonics, Bremen, Germany) in positive reflector mode in the mass range of 400–1000 Da. Two thousand laser shots were collected from each sample. External calibration was carried out using a mixture of commercially available pigments. Spectra visualization and mass list determination was performed using flexAnalysis software version 3.3 (Bruker Daltonics, Bremen, Germany).

### Microsensor measurements

Oxygen profiles in the crusts at different time points after wetting were measured using a fast-responding Clark-type oxygen microsensor with a guard cathode [27], [28]. An automated microsensor setup controlled by a computer was used and microsensor positioning with 1 µm precision was done using a VT-80 linear positioner (Micos, Germany) equipped with a DC motor (Faulhaber, Germany). The oxygen microsensor had a tip diameter of 20 µm, a stirring sensitivity of <2% (i.e. the oxygen consumption of such a microsensor is so small that it is practically insensitive to stirring and can be used in stagnant media such as sediments), and a response time of t_90_<0.5 s. A two-points linear calibration of oxygen microsensor was performed using the reading in water bubbled with N_2_ (as 0% oxygen) and equilibrated with air (as 100% air saturation) [27]. The oxygen microsensor was connected to fast-responding picoampermeter and the signals were recorded through a data acquisition card (DAQCard-AI-16XE-50, National Instruments, Austin, TX, USA) on a computer.

## Results and Discussion

**Figure 1:**
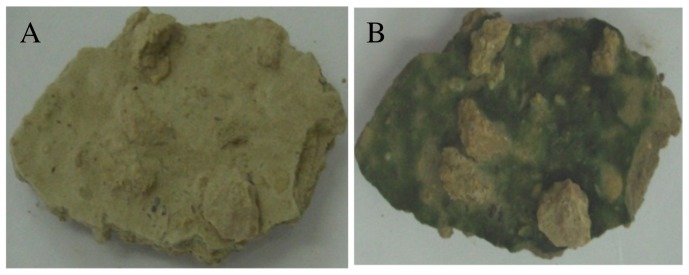
Photographs showing the soil surface colour in crust pieces before (A) and 20 minutes after addition of water (B).

**Figure 2:**
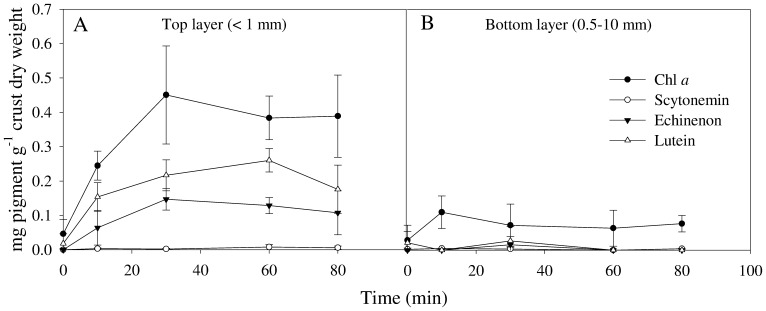
Pigment concentrations (mg g^−1^ crust) from the top (<1 mm) and bottom (1–10 mm) layers of crusts (n = 3) after addition of water at different time intervals as analyzed using high performance liquid chromatography (HPLC). Note that the pigment concentrations at T0 were obtained from extracts of crust pieces directly after wetting.

The greening of the surface in our crusts was clearly visible by bare eyes in 3–5 minutes after wetting and the colour intensified with time (Fig. 1). Cyanobacterial sequences from this crust constituted 68% of the total number of 16S rRNA bacterial sequences (i.e. 20,378 reads), most of which belonged to the genera *Microcoleus*, *Crinalium* and *Nostoc* (Fig. S1 A and B). While *Oscillatoria* sp. was found to cause the greening in crusts from Spain, high abundance of *Microcoleus vaginatus*, identified by the morphology and 16S rRNA sequences of picked filaments (Fig. S1 C and D), were concentrated in the topsoil green layer of our crusts. The greening occurred in the dark and in the light, in inverted crust pieces and always in the top layer regardless of whether the piece was wetted from top, bottom or sides. Thus, the greening was light and gravity-independent. Since cyanobacteria constitute the dominant and only oxygenic phototrophic microorganisms in these crusts, cyanobacterial pigments were quantified in the top (<1 mm) and bottom (1–10 mm) layer of the crusts to check if they move upwards after wetting. If so, they would be expected that pigments will increase in the top layer but decrease in the bottom layer due to the re-localization of cyanobacteria. HPLC analysis detected four pigments (i.e Chl *a*, scytonemin, lutein and echinone) with Chl *a* as the most prominent (Fig. 2). The concentration of Chl *a*, used as a proxy for cyanobacterial abundance, increased significantly (*P* = 0.001) in the top <1 mm layer from 0.05 to 0.25 mg g^−1^ crust already after 10 minutes of wetting, and again up to 0.45 mg g^−1^ after 30 minutes, after which it reached a plateau (Fig. 2A). Unexpectedly, the Chl *a* concentration remained low in the bottom layer, and although they slightly increased during the first 10 minutes, this increase could not account for the Chl *a* increase in the top layer (Fig. 2B). The increase in Chl *a* concentration in the top layer was not observed in autoclaved crusts, indicating that this increase is related to the recovery of viable cyanobacteria. This experiment was repeated several times and the same pattern was reproducibly obtained in all crust pieces. The observed increase in pigments was even observed in crust pieces that had a top layer as thin as 0.5 mm. When the crust pieces were left to desiccate under field conditions, detectable Chl *a* concentration by HPLC started to decrease after 1–3 days, depending on the speed of desiccation. Although cyanobacteria was shown to migrate a distance of 1.5–2 mm in other crusts (12, 21), our observation suggested that cyanobacteria in our crusts did not move from deeper layers to the surface and the greening was due to the rapid recovery of pigments.

**Figure 3:**
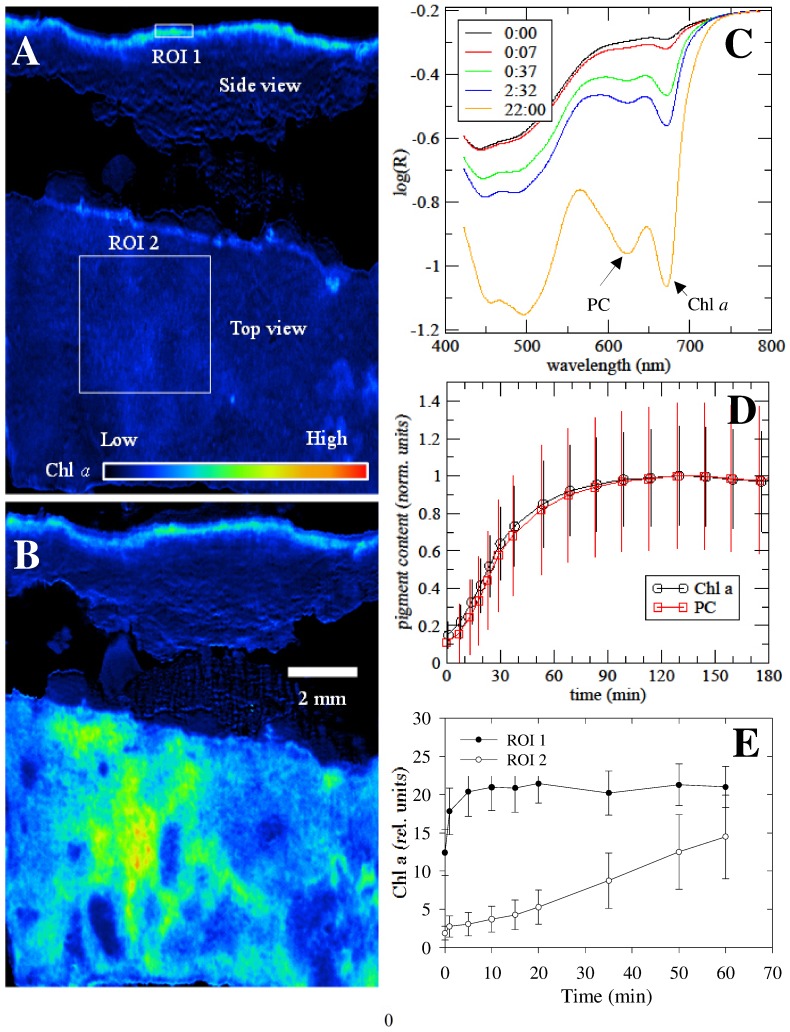
Spectral imaging showing the reflectance variation of a crust surface before (A) and 60 minutes after addition of water (B) in vertically and horizontally positioned crust pieces. Chl *a* and PC indicate the spectral signals for chlorophyll *a* and phycocynin absorption, respectively at different time intervals (indicated in hours in the insert) after addition of water (C). The change of these two pigments during a wetting period of 180 minutes is shown (D). The concentration of Chl *a* showed an increase with time in crust pieces monitored from the top and from the sides (E).

To confirm this, crust pieces were imaged from the top and from the side with a hyperspectral camera [25] (Fig. 3A and 3B), in order to localize the exact depth at which cyanobacteria reside in crusts, to monitor pigment recovery and to directly visualize the active movement of cyanobacteria, if any. Imaging of the crust from the top showed that the concentration of both Chl *a* and phycocyanin instantly increased after wetting and this increase continued up to ca. 90 minutes, after which it remained approximately stable (Fig. 3A–E). This dynamic is congruent with that obtained by HPLC. The concentrations of the two pigments increased markedly with time, especially when the crust pieces were kept wet for several days (Fig. S2 and S3). This phenomenon is due to cyanobacterial growth. Imaging of the vertical section of the crusts showed that cyanobacteria mainly reside in a 0.2–0.3 mm close-packed layer located at 0.2 mm from the surface (Fig. 3A). While Chl *a* and phycocyanin concentrations in this layer also increased after wetting, their vertical distribution did not change and this layer did not move towards the surface in the course of wetting (Side view in Fig. 3A and 3B). This confirms the conclusion based on the HPLC data that greening of the crust surface upon wetting was not due to cyanobacterial hydrotaxis.

**Figure 4:**
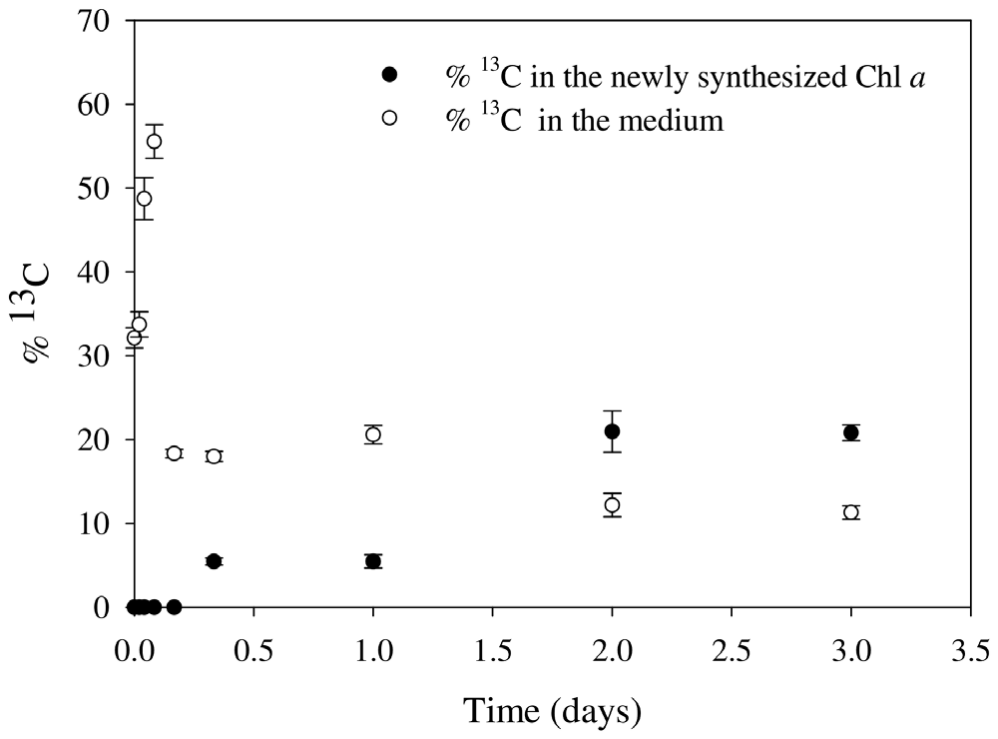
The percentage of ^13^C label in the newly synthesized Chl *a* determined using MALDI-TOF mass spectrometry and the decrease of ^13^C labeled bicarbonate in the medium (n = 3).

The increase in the concentrations of Chl *a* within minutes after addition of water to crusts, as could be assessed by both HPLC and hyperspectral imaging, raised the question whether this molecule was recovered from pre-existed precursors or was synthesized *de novo*. To address this question, ^13^C labeling experiments were performed and the accumulation of the ^13^C-label in the Chl *a* molecules was assessed by MALDI-TOF mass spectrometry. Several peaks that corresponded to ^12^C labeled molecules of Chl *a* were detected in all crusts, even in dry samples (Fig. S4). The intensity of these peaks increased with time, confirming the previously observed increase in Chl *a* concentration after wetting, however without the appearance of prominent ^13^C labeled peaks in the first 8 h (Fig. S4). ^13^C labeled peaks that corresponded to newly synthesized Chl *a* became prominent only after 48 h (ca. 20% label) of incubation (Fig. S4 and Fig. 4). Further evidence for the incorporation of ^13^C into Chl *a* molecules was the observed decrease in the concentration of ^13^C bicarbonate in the medium (Fig. 4). The ^13^C labeling experiments clearly demonstrated that Chl *a* was already present in crusts and rapidly recovered after hydration and the *de novo* synthesis of Chl *a* was only detectable when cyanobacteria in crusts started to grow.

**Figure 5:**
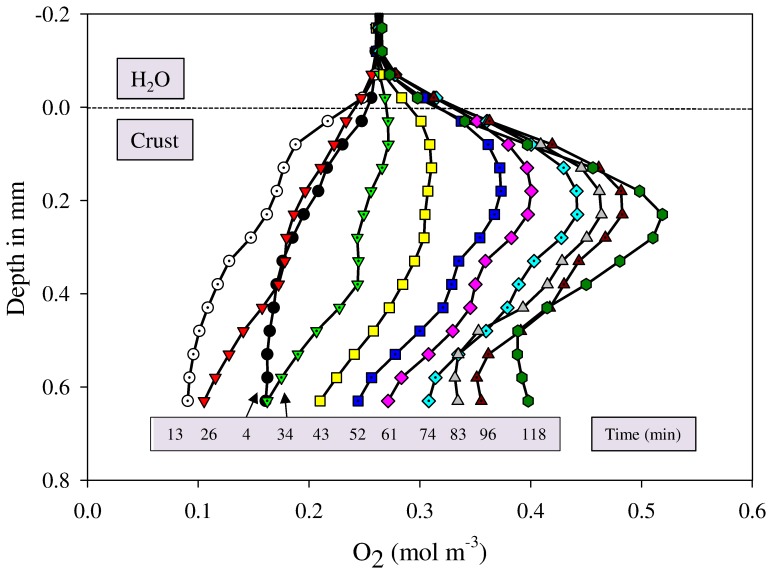
Net oxygen production and recovery of respiration and photosynthesis was monitored in the wet crust piece using oxygen microsensor measurements. The time after wetting is indicated at the bottom of each profile. Note that respiration started within 4 minutes and increased up to 13 minutes, after which oxygen was produced via photosynthesis and net oxygen production reached a maximum after 118 minutes. The oxygen profiles did not shift upward and oxygen maxima stayed at the same depth, indicating the absence of upward migration of cyanobacteria.

Microsensor measurements using oxygen electrodes were performed to find out if the increase in pigment concentrations was concomitant with a quick recovery of the crust's photosynthetic activities and to check again for any movement of cyanobacteria. Vertical shifting of the O_2_ maxima has been previously used as a proof of upward migration of cyanobacteria in response to salinity (i.e. halotaxis) [16] and UV radiation [17]. Oxygen consumption inside the crust started within the first 4 minutes after water addition and increased with time, as indicated by the shape of the profile at 13 minutes (Fig. 5). After ca. 15 minutes of wetting, the net oxygen production progressively increased, indicating higher rates of photosynthesis than respiration and an oxygen peak appeared at a depth of 0.2–0.4 mm (Fig. 5). Steady state oxygen profiles were reached after 118 minutes and the calculated net photosynthesis from these profiles was ca. 92 µmol m^−2^ h^−1^. Previous studies also showed similar quick recovery of microbial processes in desiccated species of cyanobacteria and in intact crust pieces, with respiration starting immediately within seconds and photosynthesis within minutes to few hours, followed by nitrogen fixation within 2–6 h [11], [22], [29], [30]. During the period of 118 minutes of wetting, the oxygen peaks remained at the same depth (Fig. 5), indicating that cyanobacteria did not change their position during the wetting period.

All techniques used in this study congruently demonstrated that the active hydrotactic movement of cyanobacteria towards water as a stimulus in the Omani crusts is implausible. While cyanobacteria were located between 1.5 and 2.1 mm deep in the crusts from Spain, where hydrotaxis occurred [21], they reside very close to the surface of our crusts at a depth of 0.2 mm. Since our crusts were collected from underneath the canopy of an *Acacia tortilis* tree, the shade provided by this tree could provide enough protection from UV that the cyanobacteria can remain on the soil surface, in contrast to the unprotected crusts used by others. Because of the presence of cyanobacteria at the surface in our crusts, little amounts of added water would immediately reach them. Furthermore, water is transported very fast by capillary action in crusts, especially in our crusts, which possess a loamy sand texture [22]. Previous studies have demonstrated that the hydrotactic movement of cyanobacteria is an energy-requiring process [12], [21]. This means that cyanobacteria cannot move unless they are viable and metabolically active. Since cyanobacteria are dormant in the desiccated state, active movement of cyanobacteria is only possible after they become hydrated and resume their respiration activities. This assumption is consistent with our microsensor measurements, which showed that respiration was the first microbial process to recover after rewetting. In spite of that, it is possible that the tightly bound filaments of *Microcoleus vaginatus* at the crust's surface relax and swell in the presence of water leading to a slight increase in the thickness of the cyanobacterial layer. Moreover, cyanobacteria might exhibit a phototactic movement in order to capture more light for their photosynthetic activities, but only after hydration and production of energy via respiration. Such movement has been indeed observed in benthic biofilm communities from a saltmarsh after rewetting [31].

The quick restoration of pigments and photosynthetic activities have been previously described in desiccated plants, mosses, lichens as well as cyanobacteria [32]–[35]. Chl *a* and its precursors were apparently retained in the desiccated cells since *de novo* synthesis was only possible after growth of cyanobacteria. Although Chl *a* molecules could be *de novo* synthesized using the already existing C storage compounds accumulated when cells are exposed to desiccation [15], we believe that this is unlikely to occur within 5 minutes given that chl *a* is a complex molecule and its synthesis involves several enzymatic steps [36]. The detection of low concentrations of intact Chl *a* molecules immediately after addition of water (T = 0 in Fig. 2 and 3) and the significant increase in their concentration after 5 minutes of wetting suggest that these molecules were either masked or only their precursors existed in the desiccated cells. Previous research on phototrophic organisms showed that, under harsh environmental conditions, Chl *a* degrades into several intermediates including colourless compounds [37]–[41]. The degradation steps involve either the loss of the magnesium from the centre of the molecule or the loss of the phytol ring [37]. Further degradation of Chl *a* results in the production of a number of distinct phaeophytins, chlorophyllides and phaeophorbides. The reassembly of Chl *a* molecule after water addition can be achieved by the addition of the magnesium ion or the phytol tail to these intermediate degradation products. Indeed, a substantial part of chlorophyllide and phytol, released during chlorophyll degradation in *Synechocystis* sp. PCC 6803 were shown to be recycled for the biosynthesis of new chlorophyll molecules [41]. Carotenoids (i.e. echinenon and lutein) were apparently also preserved in the desiccated cyanobacteria. Carotenoids contribute significantly to the protection of the photosynthetic machinery from oxidative damage by acting as sunscreen pigments and antioxidants through quenching of singlet oxygen, releasing excessive energy and radical scavenging [42]. Scytonemin exhibited a much slower recovery pattern than Chl *a* and carotenoids. This indicates that cyanobacteria probably allocated most energy to recover pigments that are more relevant to the restoration of their photosynthetic activities. Previous research has demonstrated that the recovery and synthesis of scytonemin is dependent on exposure to UV radiation and can take more than two days [43], [44].

Although chlorophyll and its precursors are known to be sensitive to photooxidation when extracted, irreversible photooxidation damage seems to be rare when these molecules are retained in desiccated organisms. For instance, the desiccation tolerant cyanobacterium *Nostoc commune* has been shown to retain its pigments over 100 years of desiccation in a herbarium collection and revive immediately after water addition [45]. Pigments were even found preserved in silicified Proterozoic stromatolites [46]. This suggests that these molecules are extremely stable and remain largely intact in desiccated cells. Reduced degradation of these molecules under desiccation conditions could additionally be attributed to limited heterotrophic microbial activities. Using chlorophyll fluorescence analysis, it was shown that desiccated mosses, lichens and even cyanobacteria protect their photosynthetic apparatus from photooxidation by reducing the ground chlorophyll fluorescence (*F*′) to almost zero, a process known as fluorescence quenching [32], [47], [48]. This process was recently described in intact desiccation tolerant cyanobacterial crusts from China [49].

We conclude that desiccation-tolerant cyanobacteria in crusts have developed unique mechanisms to survive drying and wetting episodes. Their photosynthetic apparatus remain essentially intact and return to a functional state with remarkable speed. In the Omani crusts, cyanobacteria did not exhibit any hydrotactic movement to track water but instead increased their Chl *a* production and restored their photosynthetic activities within minutes of water addition.

## Supporting Information

Figure S1 **Relative abundance of the most common bacterial classes (A) and genera (B) encountered by pyrosequencing of a crust sample.** Note that cyanobacteria constitute 68% of the total number of sequences (i.e. 20,378 reads) and sequences belonging to the genus *Microcoleus* were the most abundant. Picked cyanobacterial filaments form the crust's surface after wetting were analyzed using direct microscopy (C) and 16S rRNA-based phylogeny (D). These filaments were identified as *Microcoleus vaginatus*.
(TIFF)

Figure S2 **The progressive change in Chl** ***a*** **concentration was clearly detectable by hyperspectral imaging (C-F).** Time after wetting is indicated on top of the images.
(TIFF)

Figure S3 **The drastic increase in Chl** ***a*** **concentration in crust pieces after 1200 minutes of wetting due to cyanobacterial growth.**
(TIFF)

Figure S4 **MALDI-TOF mass spectra representing Chl** ***a*** **peaks at different time points (0, 8, 24 and 48 hour) after addition of water.**
(TIFF)
