# Supplementary figures and images for: Rapid Recovery of Cyanobacterial Pigments in Desiccated Biological Soil Crusts following Addition of Water
Source: PLoS One. 2014 Nov 6;9(11):e112372. doi: 10.1371/journal.pone.0112372 (PMC4223047; doi:10.1371/journal.pone.0112372)

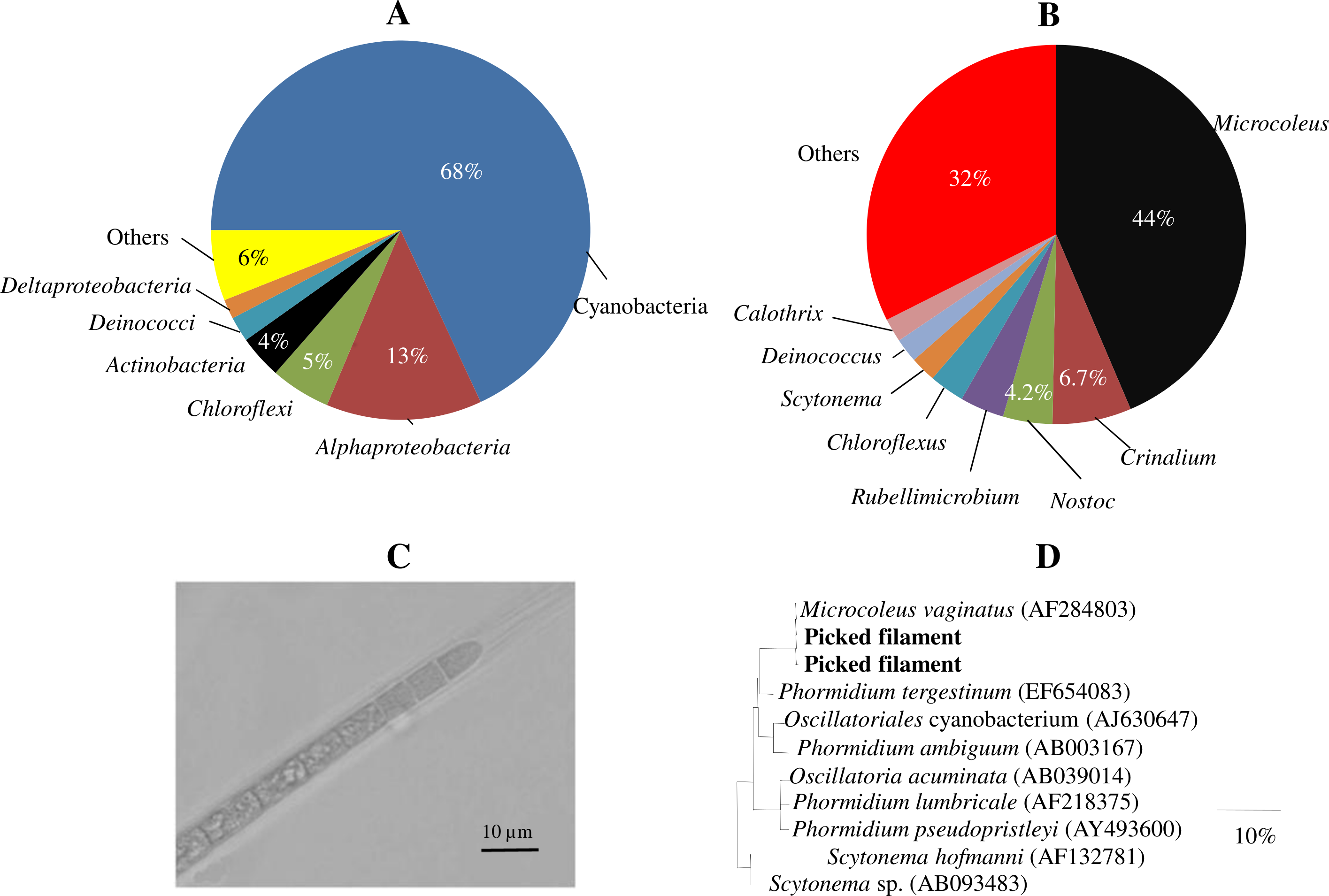

Supplement: Figure S1 — Relative abundance of the most common bacterial classes (A) and genera (B) encountered by pyrosequencing of a crust sample. Note that cyanobacteria constitute 68% of the total number of sequences (i.e. 20,378 reads) and sequences belonging to the genus Microcoleus were the most abundant. Picked cyanobacterial filaments form the crust's surface after wetting were analyzed using direct microscopy (C) and 16S rRNA-based phylogeny (D). These filaments were identified as Microcoleus vaginatus. (TIFF) [file pone.0112372.s001.tiff]

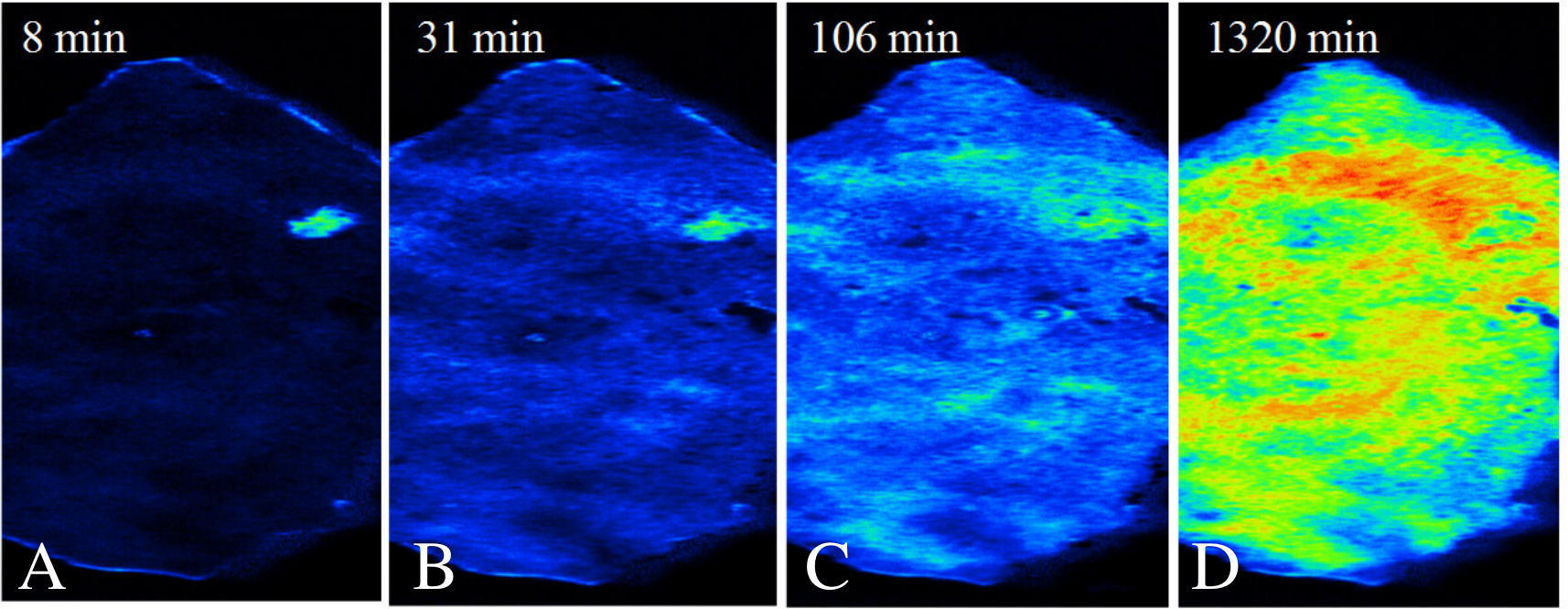

Supplement: Figure S2 — The progressive change in Chl a concentration was clearly detectable by hyperspectral imaging (C-F). Time after wetting is indicated on top of the images. (TIFF) [file pone.0112372.s002.tiff]

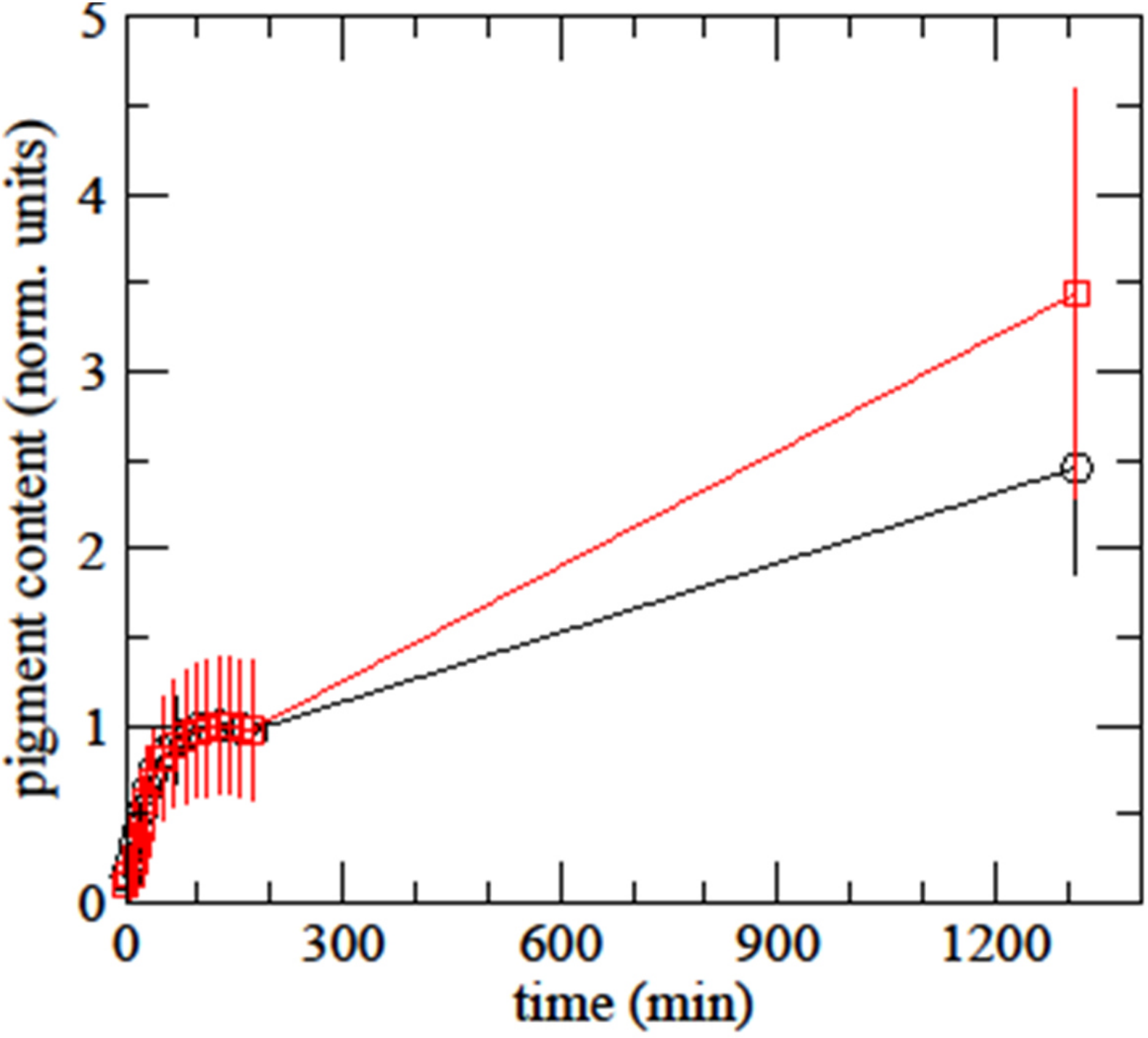

Supplement: Figure S3 — The drastic increase in Chl a concentration in crust pieces after 1200 minutes of wetting due to cyanobacterial growth. (TIFF) [file pone.0112372.s003.tiff]

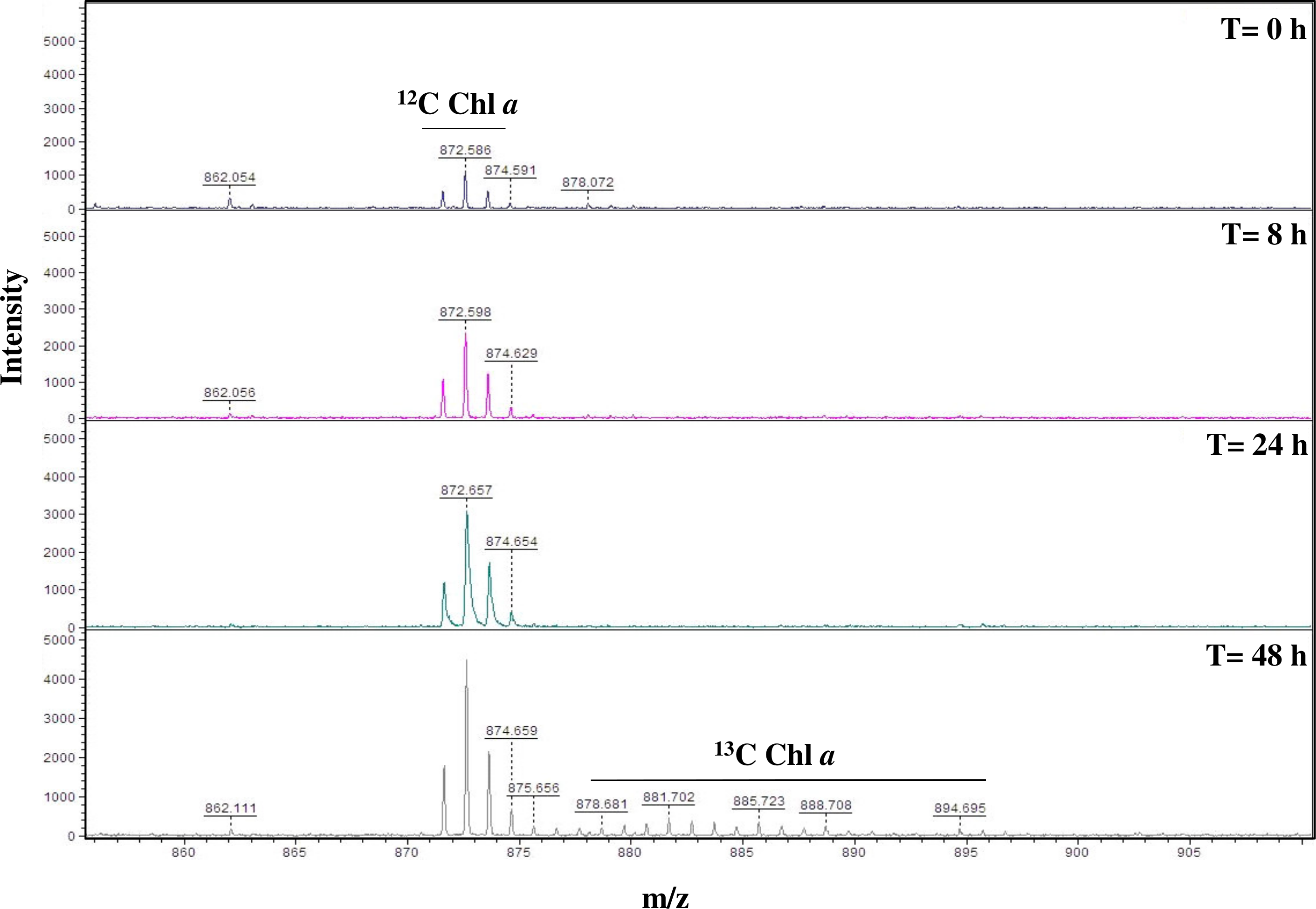

Supplement: Figure S4 — MALDI-TOF mass spectra representing Chl a peaks at different time points (0, 8, 24 and 48 hour) after addition of water. (TIFF) [file pone.0112372.s004.tiff]
